# Drivers and Inhibitors in the Acceptance of Meat Alternatives: The Case of Plant and Insect-Based Proteins

**DOI:** 10.3390/foods9091292

**Published:** 2020-09-14

**Authors:** Wim de Koning, David Dean, Frank Vriesekoop, Luis Kluwe Aguiar, Martin Anderson, Philippe Mongondry, Mark Oppong-Gyamfi, Beatriz Urbano, Cristino Alberto Gómez Luciano, Bin Jiang, Wendy Hao, Emma Eastwick, Zheng (Virgil) Jiang, Anouk Boereboom

**Affiliations:** 1 Faculty of Agribusiness and Commerce, Lincoln University, Lincoln 7647, New Zealand; Wim.deKoning@lincoln.ac.nz (W.d.K.); David.Dean@lincoln.ac.nz (D.D.); 2 Department of Food Technology and Innovation, Harper Adams University, Newport TF10 8NB, Shropshire, UK; ldeaguiar@harper-adams.ac.uk (L.K.A.); manderson@harper-adams.ac.uk (M.A.); wendyhao315@hotmail.com (W.H.); eastwickemma@gmail.com (E.E.); Virgiljiangzheng@163.com (Z.J.); aj.boereboom@gmail.com (A.B.); 3 Department of Food Technology, HAS University of Applied Science, 5223 DE Den Bosch, The Netherlands; 4 USC 1422 GRAPPE, INRAE, Ecole Supérieure d’Agricultures, SFR 4207 QUASAV, 49000 Angers, France; p.mongondry@groupe-esa.com (P.M.); mogyamfi2@gmail.com (M.O.-G.); 5 Department of Agricultural and Forestry Engineering, University of Valladolid, 47002 Valladolid, Spain; beatriz.urbano@uva.es; 6 Department of AgriBusiness Engineering, Specialized Institute of Higher Studies Loyola, 91000 San Cristóbal, Dominican Republic; cristinoalbertogomez@gmail.com; 7 College of Bioengineering, Beijing Polytechnic, Beijing 100176, China; jiangbin@bpi.edu.cn

**Keywords:** willingness to try, neophobia, structural equation model

## Abstract

Insects as an alternative protein source has gained traction for its advantageous environmental impact. Despite being part of many traditional food cultures, insects remain a novelty in Western cultures and a challenging concept for many. Even though plant-based protein alternatives are not facing the same barriers, product unfamiliarity and limited exposure hinder adoption, which could be detrimental to growth within the food sector. This study is aimed at evaluating plant- and insect-based proteins as alternative dietary proteins. A model indicating the drivers of consumer attitudes towards meat-alternative proteins and consumer willingness to try, buy, and pay a premium was tested. Further, 3091 responses were collected using surveys in nine countries: China, USA, France, UK, New Zealand, Netherlands, Brazil, Spain, and the Dominican Republic. Structural Equation Modelling was used to analyze the data. We found that consumer’s behavioral intentions towards both plant-based and insect-based alternatives are inhibited by food neophobia but to an extent, are amplified by the perceived suitability and benefits of the protein, which in turn are driven by nutritional importance, environmental impact, healthiness, and sensory attributes for both alternatives. The expectation of the nutritional value of meat is the strongest (negative) influence on perceived suitability/benefits of plant-based protein and willingness to try, buy, and pay more for plant-based proteins, but it only has a relatively small impact on the suitability/benefits of insect-based protein and no impact on willingness to try, buy, and pay more for insect-based proteins. Overall, we conclude that consumer adoption towards meat alternatives is complex and is strengthened by the perceived suitability/benefits of the protein and general importance of perceived food healthiness and sustainability. Conversely, adoption is hindered by dietary factors and the experiential importance of meat and food neophobia.

## 1. Introduction

Much has been publicized about how the unsustainable ways of traditional meat production and consumption [1,2,3] is detrimental to both the environment and human health [4,5,6]. As a result, meat, particularly red meat, has attracted much criticism in recent years [7,8]. This, compounded with demographic pressures and socio-economic growth trends, has encouraged new product development and the introduction of a variety of alternatives to traditional animal proteins, thus extending the availability of meat substitutes in many markets. In order for the necessary changes to become a reality in our current food systems, we need to have a better understanding of how consumers view meat alternatives and gauge their willingness to change their purchasing and consumption habits.

Meat alternatives are not new, particularly plant-based and mycoproteins such as Quorn. Nevertheless, Quorn’s global market share has not spread much outside the UK and the product has remained niche in most countries outside the UK. Conversely, pulses, which are a great source of plant proteins, have been a traditional part of staple diets in many cultures for millennia [9]. More recently, plant-based protein sources have been part of extensive new product development by the food industry bringing meat alternatives to the market that are promoted under higher sustainability credentials [10,11], thus catering to the burgeoning vegetarian and vegan segments.

The potential of insects as an alternative protein source has also gained traction because of advantages in resource usage, such as land, feed, water, and energy, and the role they can play in circular production systems [12,13]. Insects are and have been part of food cultures of large swathes of the world population. Yet, it is felt that in the more economically developed western countries, insect eating, entomophagy, and the consumption of products made with insect protein are still a novelty and a challenging concept for many consumers. Consumers’ unfamiliarity and limited exposure to different food products hinders the adoption of new foods, which holds true for most foods made with alternative proteins [14,15].

### 1.1. Theoretical Underpinning

Western consumers tend to possess an ingrained barrier to eating insects and insect-based products, which is expressed through fear and disgust [16]. Such a behavior is typical of a food neophobic trait. Kush et al. [13] posited that consumers tended not to change their purchasing behaviors easily. The consumers’ reluctance to change could be attributed to an inbuilt evolutionary-derived encoded instinct to protect humans against potential poisonous foods over familiar ones that are more beneficial to health and growth [17,18]. Thus, a predisposition to avoid unusual foods is based on instinctual neophobia [19], which has been socially constructed and filtered through the consumers’ system of values [20]. This could play a major role with regards to protein consumption, where an aversion to alternative proteins could constitute a major impediment for replacing meat for another substitute because of the consumer’s values, dietary habits, and preferences [21]. This is not unlike when plant-based proteins were first introduced into people’s diets more widely [22].

Some of this behavior can be described as food neophobia, which is considered an expression of an aversion trait in consumers’ choice behavior with regards to new foods [23]. However, the more frequent and intense the exposure to a new food product through information, education, and experimentation, the lesser the rejection by consumers. Therefore, it can be argued that food neophobia boundaries can be shifted over time. Clark and Bogdan [24] demonstrated that considerable barriers continue to confront the expansion of the market for plant-based proteins. However, their research suggested that once consumers have adopted plant-based meat alternatives, they were more likely to try new plant-based protein versions within the same product category in the future.

Schouteten et al. [25] compared meat, plant, and insect protein in the format of burgers. The overall liking of the plant and insect burger was similar, however the majority of consumers expressed disappointment for both alternatives compared to the traditional meat burger. However, when informed of the ingredients prior to tasting, the approval of the insect burger was significantly higher compared to when the information was not disclosed [25]. Gómez-Luciano et al. [26] found a greater willingness to purchase plant-based protein compared to insect-based proteins, however the responses varied between countries analyzed. Despite a reluctance to immediately adopt new foods, consumers indicated to being open to future changes, supporting a growing dietary shift to alternative dietary proteins [27]. These findings are in agreement with van der Weele et al. [28] who concluded that organizational and institutional coordination were required to enable the acceptance of meat alternatives (insect, pulses, and cultured meat), with recommendations to drive nutritional, sustainability, technological, and societal changes.

It is well understood that one of the major constraints concerning consumers’ willingness to engage with sustainable food innovations is the consumers themselves [25,26,27]. Pliner and Hobden [29] developed a Food Neophobia Scale (FNS), which has since served to measure the consumers’ willingness to consume foods that they might not be familiar with or have held a life-long aversion to. Cox and Evans [30] investigated food-related neophobia one step further and considered the possible aversion to new foods produced by novel technologies, which has been coined as Food Technology Neophobia [30]. Both the Food Neophobia scale and the Food Technology Neophobia scale have been widely validated in many different contexts [23,31,32]. However, Bäckström et al. [32] mentioned that familiarity played an important role in people’s willingness to try a product that they do not recognize or have not encountered before. Consequently, unfamiliar products would face barriers to consumption as they clash with habit-bound consumer behavior [32]. Capitanio et al. [33] concluded that the aversion to consume novel foods was driven by a fear of what a food product could contain regarding ingredients and the processes used in its production. Chang et al. [34] argued that for organic foods, when too much processing had taken place, a product’s perceived authenticity would be diminished, resulting in a lower purchase intention, which agrees with Eyhorn et al. [35]. Furthermore, despite a greater willingness to try a novel food product, consumers’ intentions to pay more for meat alternatives is often low [36,37]. Therefore, despite the growing literature around the topic, there is still the need to investigate the drivers that influence consumers’ attitudes towards meat alternatives. This study’s contribution is to bring to light what consumers’ attitudes would be toward willingness to buy, willingness to try, and willingness to pay a premium for meat alternatives such as plant- and insect-based products.

### 1.2. Model Development

The overarching aim of this study was to evaluate whether plant- and insect-based proteins could be realistic meat alternatives from the consumers’ point of view. In order to test a theoretical model, attitudes towards the two types of meat substitutes were analyzed and the extent to which there were differences in consumers’ attitudes and preferences between the alternatives was tested. Meat functioned as the default to which consumers could compare a widely accepted meat alternative (plant-based) and a meat alternative that could be integrated into a circular production system (insect-based) [38,39]. It also aimed at establishing a model indicating the drivers of consumers’ attitudes towards meat-alternative proteins and consumers’ willingness to try, buy, and pay a premium for them.

**Figure 1:**
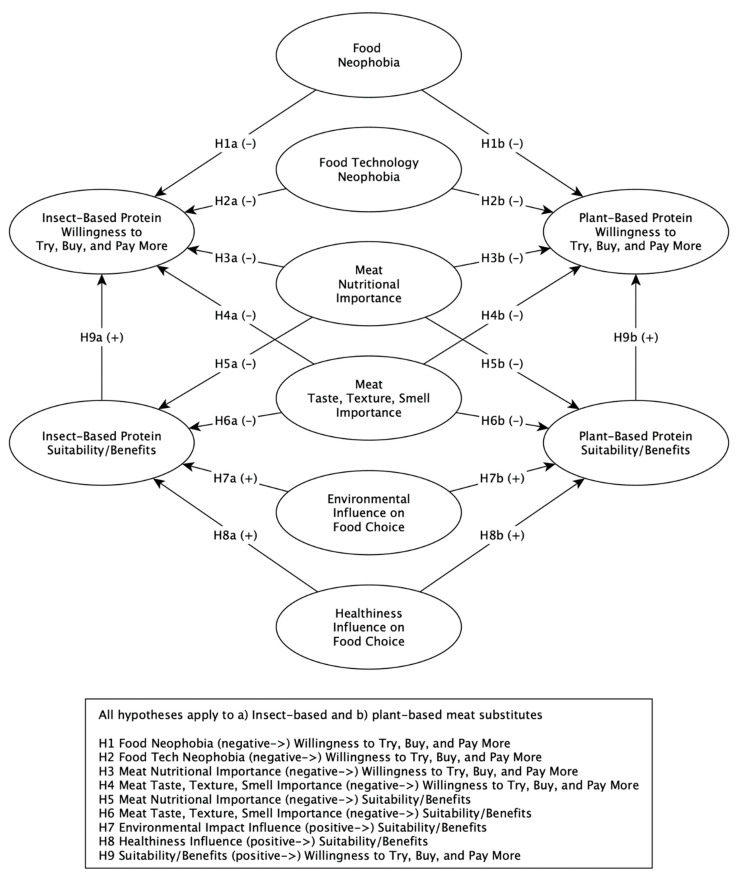
Conceptual Model and Hypotheses.

The model (Figure 1) was designed based on the literature that supported the notion that new and unfamiliar foods affected consumer behavior [20,31]. It was expected that Food Neophobia and Food Technology Neophobia would inhibit consumers’ willingness to try, to buy, and pay more for meat-alternative proteins. Nine hypotheses were tested (Figure 1). The consumers’ attitudes towards the importance of meat taste, texture, smell, and the nutritional importance of meat were expected to be negatively influenced by their perception of meat-alternative suitability and benefits [19,21]. Suitability and benefits were defined as a combination of sensory benefits, nutritional importance, environmental impact, and health influence that was unique to the meat substitute in question. The importance consumers placed on healthiness and the environmental impact of their food choices, in general, was likely to enhance their assessment of meat substitutes [40]. Consumers’ attitudes towards the suitability of and benefits derived from a particular meat-alternative protein should also augment their willingness to adopt it [26,27]. Therefore, the proposed model should establish a better understanding of how consumers viewed meat alternatives and their willingness to change their purchasing and consumption habits.

## 2. Method

A sample of 3091 responses in total was obtained from surveys carried out in nine countries. The sample was composed of 571 respondents from China (CN), 539 from the USA (US), 484 from France (FR), 366 from the UK, 268 from New Zealand (NZ), 231 from the Netherlands (NL), 216 from Brazil (BR), 210 from Spain (ES), and 206 from the Dominican Republic (DR). Data collection started in February 2017 and finished in April 2018 in CN, the US, FR, the UK, BR, ES, and the DR. From February 2018 until May 2019, data were collected in NZ and the NL. The gender distribution was 59.2% females, 38.9% males, and 1.9% who preferred not to answer. The mean age of the sample was 34, with quartile ranges of 16–21, 22–28, 29–44, and 45–86. Table 1 provides a country-by-country insight into the demographics of the survey respondents.

The questionnaire was initially written in English and then translated into the various respective languages by native speakers who were fluent in both English and their mother tongue to improve the accuracy of meaning and avoid misunderstandings by the various linguistic cohorts. The languages were also adjusted for variations in grammar/spelling, i.e., UK-English, US-English, and NZ-English; ES-Spanish and DR-Spanish; as well as Brazilian Portuguese. The translated versions were back-translated into English to ensure that the meaning had not deviated from the initial word concept or idea. The various collaborators and co-authors were responsible for distributing the survey at a country level (mainly through social media and existing e-mail contact lists). All data gathered were centrally collected and collated at Harper Adams University (HAU) in the UK. In most instances, the questionnaire was distributed in a digital format, however when requested, a hardcopy version was also made available. In the DR, the responses were predominantly collected using a hardcopy, catering for the relatively scant access to the Internet in that country. The research and questionnaire were approved by the Harper Adams University (HAU/UK) Research Ethics Committee (HAU-0006-201701). Furthermore, as part of the ethics declaration, each questionnaire also included a contact e-mail at HAU, so that questions arising from answering the questionnaire could be addressed.

### Questionnaire and Scaling

The questionnaire included various distinct sets of questions and statements consistent with a previous study [26]. The participants gave their informed consent to partake in the survey. The first group of statements probed the respondents’ attitudes towards new foods, new food technologies, health, convenience, and the environmental impact of their food choices (Table 2). More specifically, the following scales were used in the questionnaire to measure the various constructs: Food Neophobia Scale, with 10 items, adapted from Pliner and Hobden [29] (Table 2, 08.1 through to 08.10); Food Technology Neophobia Scale, with five items, which was inspired by Cox and Evans [30] (Table 2, 09.1 through to 09.8); Healthiness of Food Choices, with three items, adapted from the “impact of the healthiness of food choices” scale [40] (Table 2, 10.1 through to 10.3); and Environmental Impact of Food Choices, with three items, adapted from the “environmental impact of food choices” scales in Roberts [41] and Verbeke [37] (Table 2, 12.3 through to 12.3). Many of the above-mentioned scales were adapted from previously described tools [26,29,30,37,40,41] in relation to assessing people’s willingness to engage with new foods. In these adaptations, we made careful choices with regards to which survey items to include in our study to avoid unnecessary duplication, utilize the most appropriate items, and avoid potential survey fatigue. For instance, the original food technology neophobia scale [30] contains items covering health and environmental factors, however we found that these topics were better addressed using the survey items used elsewhere [37,40,41]. As such, we also detached those sub-topics from the original scale and addressed them separately. The second group of statements probed the respondents’ perceived importance of meat in terms of its nutritional benefits and sensory experience (Table 2). More specifically, a 3-item scale measured Meat Nutritional Importance (Table 2, 13.1 through to 13.3) and a 3-item scale measured Meat Taste, Texture, and Smell Importance (Table 2, 14.1 through to 14.3). All the questions were presented in the form of statements to which the respondents expressed their opinion using a five-point Likert scale ranging from “strongly disagree” to “strongly agree” (Table 2).

The questionnaire then included descriptions of plant-based and insect-based alternatives to meat proteins. Consumers were asked about their perceptions of the suitability of or the benefits derived from plant-based and insect-based proteins. These questions consisted of six items measuring healthiness, safety, nutrition, sustainability, taste, and affordability relative to meat protein (Table 2). Finally, a consumer behavioral intention scale was used to measure aspects such as willingness to try, willingness to buy, and willingness to pay more for plant-based and insect-based proteins. The questionnaire also collected some demographic characteristics of the respondents.

## 3. Analysis

A two-step Structural Equation Modelling was used. The first step was related to the evaluation of the measurement model using confirmatory factor analysis. This step evaluated the measurement scales and their items, examining construct convergent and discriminant validity and reliability. The second step tested the model, assessing the significance of the hypothesized relationships between the variables and confirming that goodness-of-fit criteria were satisfied. This two-step analysis was selected due to its appropriateness in the measurement and examination of structural models and testing coefficient paths. For an excellent discussion on the ongoing development and generally accepted process for employing the type of Structural Equation Modelling used in this research, see [42].

### 3.1. Construct Validity and Reliability

Construct validity was evaluated using factor loadings and average variance extracted (AVE). As shown in Table 2, the result of convergent validity assessment indicated that except for the Food Neophobia scale item, “Some foods look too weird to eat,” all of the standardized loadings were above the cut-off level of 0.5, as set by Anderson and Gerbing [43]. Except for the Food Neophobia and Food Tech Neophobia scales, Table 2 also shows that the AVE of all the scales was higher than the 0.5 cut-off level as suggested by Hair et al. [44]. Unfortunately, the removal of any items to those scales resulted in the lowering of Cronbach’s Alpha and Composite Reliability values, so it was decided not to take remedial action.

Table 2 also shows that the scales demonstrated adequate reliability. All but one (Environmental Impact Influence) of the scales had Cronbach’s Alpha values above the cut-off level of 0.7 and all the scales had composite reliability values above the suggested cut-off level of 0.7 [44].

The discriminant validity of the construct scales was acceptable using both the Fornell-Larker criterion and the Heterotrait-Monotrait (HTMT) ratio methods. Table 3 shows that the Fornell-Larcker criterion was satisfied as the shared variance between the constructs was lower than the variance captured by the construct (along the diagonal). The HTMT ratio was also satisfied as the HTMT correlation estimates between the scales were below the recommended threshold of 0.85 [45], confirming adequate discriminant validity.

### 3.2. Structural Model

Following Hair et al. [44], a bootstrapping method with 500 repetitions was applied to assess the significance of the indicator weights and the path coefficients. In addition, the corrected *R*^2^ of all constructs was estimated as a diagnostic tool to evaluate the model fit. The Goodness of Fit (GoF) measure applies the geometric mean of the communality and the average *R*^2^ for endogenous dependent constructs. The standard for evaluating the outcomes of the GoF analysis is small (0.02), medium (0.25), and large (0.36) [44]. In this research, a GoF value of 0.390 (see Table 4) shows that the proposed model of the relationship between consumer food attitudes and their assessment of and willingness to try and purchase plant-based and insect-based proteins is large, signifying that the model performs well.

Chin et al. [46] argued that an investigator should be able to employ the magnitude of *R*^2^ and Stone-Geisser’s *Q*^2^ value as a criterion for the predictive relevance of a model for a particular construct. The results of *Q*^2^ calculations for all the endogenous constructs were greater than zero, indicating that they have satisfactory predictive relevance [44].

Further, Table 4 depicts some results from testing the structural model, indicating that the model does a good job of explaining the variance of willingness to try, buy, and pay more for both meat substitutes. The model explains 33.1% (*R*^2^ = 0.331) of the variance of Plant-base willingness and 31.0% (*R*^2^ = 0.310) of the variance of Insect-based willingness. However, the model was able to explain 24.3% (*R*^2^ = 0.243) of the variance of consumer perceptions of plant-based suitability/benefits compared with only 4.2% (*R*^2^ = 0.042) of the insect-based protein suitability/benefits.

## 4. Results and Discussion

### 4.1. Food Neophobia

Food neophobia inhibits willingness to adopt both meat substitutes (Table 5), fully supporting hypotheses H1a/b, but food tech neophobia only inhibits willingness to adopt for plant-based substitutes, supporting H2b.

Faccio and Fovino [19] made it very clear in their review that the relationship between neophobia and technological innovation in the agrifood industry was complex and required nuance when the concept of neophobia was used outside its original context. Their contention was that a consumer’s willingness to try new or unusual food was filtered through their system of norms and values and until new foods or processes become more mainstream, some resistance or avoidance is expected. Our results show that Food Neophobia and Food Technology Neophobia would inhibit consumer willingness to try, buy, and pay more for meat-alternative proteins, however the notion of neophobia by itself might not have been a sufficient indicator to gauge consumers’ drivers. The possibility that for some foods, disgust could be a greater influencer than neophobia [16,18,19] should not be overlooked, however the notion of disgust itself was outside the scope of this study.

### 4.2. Perceived Importance of Meat

The results also show that meat nutritional importance only inhibited willingness to adopt plant-based substitutes (support for H3b), however meat nutritional importance negatively influenced the perceived suitability/benefits of both meat substitutes (supporting H5a/b). Meat taste/texture/smell importance inhibited willingness to adopt both meat substitutes (supporting H4a/b) and negatively influenced the perceived suitability/benefits of only insect-based substitutes (supporting H6a).

The outcome of hypotheses 3 to 6, examining attitudes towards the importance of meat taste, texture, smell, and the nutritional importance of meat, was consistent with the findings of Schouteten et al. [25] and Mishyna et al. [47].

### 4.3. Food Choice Values

The importance of the environmental impact of food choices positively influenced the perceived suitability of both meat substitutes (supporting H7a/b) and the importance of the healthiness of food choices positively influenced the perceived plant-based meat substitutes (supporting H8b).

The importance of healthiness, environmental impact, and suitability of consumers’ food choices was examined in hypotheses 7 to 9 and the results support that the food choices were clearly linked with personal values and that these determine the feasibility of a sustainable diet. This is consistent with the information about food choices influencing overall liking [24], that the role meat plays in the diet for many people is beyond its nutritional needs [48], and people rationalize meat consumption [49]. The proposed model included attitudes that were rich in moral implications linked to neophobia values, which offered a multifaceted view of how consumers viewed meat alternatives and their willingness to change their purchasing and consumption habits.

### 4.4. Behavioral Intension

Food preference research has found links between food ingredients and consumers’ willingness to try them. As such, barriers to trying unfamiliar products is linked to the absence of familiar ingredients and the requirement of a relationship between product and territorial context will determine the adoption of innovation [33]. Similarly, customers are more willing to try novel foods when they contain familiar ingredients, although they are unlikely to pay more for novel products—for example, organic meat, moderation of meat consumption, and sustainable fish are accepted, although willingness to pay more is lower than willingness to consume [36]. Furthermore, the readiness by consumers to adopt insects as an alternative meat ingredient where traditional meat consumption showed that only consumers with a weak attachment to meat would consider trying the insect alternative [37]. In this research, consumer perceptions of the suitability and benefits of insect-based meat substitutes augmented their willingness to try, buy, and pay more for them (supporting H9a). The model was able to account for 31% of the variance of behavioral intention and perceived suitability/benefits of insect-based protein was the dominant predictor of behavioral intention, with a notable non-significant influence of food tech neophobia, meat nutritional importance, and healthiness of food.

For plant-based substitutes, the model performed largely as proposed, explaining 33% of the variance of behavioral intention. The paths suggested that meat nutritional importance and plant-based suitability/benefits are the most important predictors of willingness to try, buy, and pay more for plant-based substitutes (supporting H9b).

### 4.5. Plant-Based vs. Insect-Based Comparisons

The literature [25,26,27,28] suggests that for many components of the model, plant-based meat substitutes are likely to be considered more suitable and consumers are more willing to adopt them compared to insect-based substitutes. While no specific predictions were made, Table 6 shows the Paired Sample T tests for comparisons between plant-based and insects-based examples for specific items from the suitability/benefits scales and the willingness to try, buy, and pay more scales. For every pair, the plant-based responses were significantly higher than the insect-based responses, which is most likely due to the notion that plant-based meat substitutes are well established in most cultures, while insect-based meat substitutes are still a novelty with a strong stigma attached [26,27].

Overall, we analyzed consumer perceptions with regards to meat and two alternative dietary protein sources in nine very diverse countries: China, USA, France, UK, New Zealand, Netherlands, Brazil, Spain, and the Dominican Republic. We analyzed our data (3091 respondents) as a single global cohort, rather than providing country-by-country analyses. A country-by-country analysis would have provided more granularity in interpretation; however, it would also have created a very complex and potentially confusing discussion. Our global approach to data interpretation does provide a clear insight into consumers’ perceptions regarding alternative protein sources.

## 5. Conclusions

The findings in this study clearly show that there are differences in consumer attitudes and these influence behavioral intentions towards plant-based and insect-based protein as meat alternatives. To gain more insight into behavioral intentions (willingness to try, buy, and pay a premium), a model was proposed and tested to evaluate the consumers’ attitude drivers and determine if plant- and insect-based proteins were realistic meat alternatives. This confirms that consumer adaptation towards sustainable meat alternatives can be complex and is influenced by a diverse set of attitudinal and cognitive-based perceptions.

Our results show that consumer’s behavioral intentions towards meat alternatives are inhibited by food neophobia but to a larger extent, are augmented by the perceived suitability and benefits of the protein. The perceived suitability and benefits of the protein alternatives are driven by environmental impact, healthiness, nutritional importance, and sensory attributes for both plant and insect alternatives. Food neophobia and food tech neophobia do not influence the consumer’s attitude towards suitability and benefits but have a very clear influence on the behavioral intentions and tend to decrease the willingness to try, buy, and pay more for meat-alternative proteins. The model also shows that consumer attitudes about the environmental impact and to a lesser extent, the healthiness of food, lead to stronger perceived suitability and benefits of plant-based protein. Stronger importance of meat nutrition and to a lesser extent, meat taste, texture, and smell, lead to lower levels of plant-based protein suitability and perceived benefits and lower willingness to try, buy, and pay more for plant-based proteins. For insect-based protein, consumer attitudes towards the suitability and benefits are a strong predictor of willingness to try, buy, and pay more, but those attitudes do not seem to be clearly derived from importance of healthiness, environmental impact of food in general, or their attitudes towards meat. The importance of meat nutritional value is the strongest (negative) influence on perceived suitability/benefits of plant-based protein and willingness to try, buy, and pay more for plant-based proteins, but it only has a small impact on the suitability/benefits of insect-based protein and no impact on willingness to try, buy, and pay more for insect-based proteins.

This study indicates that consumer preferences are influenced by behavioral intentions but does not consider all possible underlying individual attributes such as educational status, knowledge of food and its origins, nutritional values of meat and its alternatives, or the ability to cook a meal. Neither does it consider the potential change in those behaviors with consideration to the importance of, for example, further processing of food ingredients. The contribution of this study is evident by the model created, which is a valuable tool to evaluate what needs to change in consumer attitudes to alter their behavioral intentions. The consumer’s understanding of the nutritional role of meat in their diets and the sensory aspects of meat seem to be pivotal as they influence both attitudes and behavioral intentions.

This study is based on 3091 respondents from nine countries and did not answer the cultural role of meat consumption. Further studies should focus on whether food tech neophobia is a larger driver in more technologically advanced meat alternatives such as fungal-based protein and cultured meat. Further, it is unclear what role culture plays as a driver of consumer attitudes towards meat alternatives, such as whether meat substitutes are more accepted in low meat-eating cultures compared to high meat-eating cultures.

## Figures and Tables

**Table 1:** Demographics (gender and age) of the survey respondents per country.

Country	*n*	Gender			Age	
		Male%	Female%	Prefer not to Say	Mean ± SD	Range
China	571	38.0%	60.8%	1.2%	31.2 ± 11.6	19–72
USA	539	24.6%	75.4%	NA *	44.1 ± 21.7	18–71
France	484	59.9%	31.8%	8.3%	29.0 ± 17.3	18–68
UK	366	23.8%	76.2%	NA	32.0 ± 16.8	19–67
New Zealand	268	46.8%	53.2%	NA	37.9 ± 12.9	18–70
Netherlands	231	37.7%	62.3%	NA	29.6 ± 15.4	17–70
Brazil	216	43.1%	56.9%	NA	38.3 ± 22.1	17–77
Spain	210	49.5%	48.1%	2.4%	35.1 ± 19.5	19–83
Dominican Republic	206	32.5%	66.0%	1.5%	26.2 ± 9.5	16–69
**Total**	**3091**	**38.9%**	**59.3%**	**1.8%**	**34.1 ± 15.4**	**16–83**

* NA = not applicable.

**Table 2:** Scale Loadings, Reliabilities, and Convergent Validity.

Scales and Items	Factor Loadings	Cronbach’s Alpha	Composite Reliability	AVE
**Food Neophobia**		0.795	0.844	0.355
08.1R. I am constantly sampling new and different foods	0.576			
08.2. I do not trust new foods	0.662			
08.3R. I like foods from different countries	0.615			
08.4. If I do not know what is in a food, I will not eat it	0.523			
08.5R. At dinner parties I will try a new food	0.565			
08.6. Some foods look too weird to eat	0.485			
08.7. I am afraid to eat things I have never had before	0.673			
08.8. I am very particular about the foods I eat	0.506			
08.9R. I will eat almost anything	0.588			
08.10R. I like to try new foods from all over the world	0.717			
**Food Tech Neophobia**		0.746	0.829	0.495
09.1. The benefits of new food technologies are often grossly overstated	0.585			
09.3. There are plenty of tasty foods around so that we do not need to use new food technologies to produce more	0.745			
09.5. New food technologies decrease the natural quality of foods	0.792			
09.7R. New products using new food technologies can help people have a balanced diet	0.673			
09.8R. Innovations in food technology can help us produce foods in a sustainable manner	0.707			
**Healthiness Influence**		0.716	0.838	0.633
10.1R. The healthiness of food has little impact on my food choices	0.718			
10.2. I am very particular about the healthiness of the food I eat	0.842			
10.3R. I eat what I like and I do not worry much about the healthiness of food	0.822			
**Environmental Impact Influence**		0.647	0.810	0.588
12.1. When I buy foods I try to consider how my use of them will affect the environment	0.699			
12.2. I am worried about humankind’s ability to provide the nutritional needs for all people living on earth now	0.830			
12.3. Something drastic has to change in order to feed all the people on earth by 2050	0.766			
**Meat Nutritional Importance**		0.779	0.873	0.698
13.1. Eating meat is necessary for obtaining beneficial nutrients	0.871			
13.2. The nutritional benefits of meat can easily be matched by alternative protein sources	0.732			
13.3. Meat is an important part of a healthy and balanced diet	0.894			
**Meat Taste, Texture, Smell Importance**		0.941	0.962	0.895
14.1. The taste of meat is important to me	0.952			
14.2. The texture of meat is important to me	0.955			
14.3. The smell of meat is important to me	0.931			
**Plant-Based Protein Suitability/Benefits**		0.786	0.854	0.546
19.1. Plant-based protein is healthy	0.836			
19.2. Plant-based protein is safe to eat	0.697			
19.3. Plant-based protein is nutritious	0.840			
19.4. Plant-based protein is more sustainable	0.765			
19.6. Plant-based protein is cheaper	0.506			
**Plant-Based Protein Willingness to Try, Buy, and Pay More**		0.726	0.845	0.646
20.1. Willing to try plant-based protein	0.752			
20.2. Willing to purchase plant-based protein	0.891			
20.3. Willing to pay more for plant-based protein	0.760			
**Insect-Based Protein Suitability/Benefits**		0.890	0.920	0.699
35.1. Insect-based protein is healthy	0.907			
35.2. Insect-based protein is safe to eat	0.880			
35.3. Insect-based protein is nutritious	0.886			
35.4. Insect-based protein is more sustainable	0.830			
35.6. Insect-based protein is cheaper	0.653			
**Insect-Based Protein Willingness to Try, Buy, and Pay More**		0.823	0.893	0.740
36.1. Willing to try insect-based protein	0.915			
36.2. Willing to purchase insect-based protein	0.946			
36.3. Willing to pay more for insect-based protein	0.697			

**Table 3:** Scale Discriminant Validity.

Fornell-Larcker Criterion	Environmental Impact Influence	Food Neophobia	Food Tech Neophobia	Healthiness Influence	Insect-Based Protein Suitability/Benefits	Insect-Based Protein Willingness to Try, Buy, and Pay More	Meat Nutritional Importance	Meat Taste, Texture, Smell Importance	Plant-Based Protein Suitability/Benefits	Plant-Based Protein Willingness to Try, Buy, and Pay More
Environmental Impact Influence	0.767									
Food Neophobia	−0.102	0.595								
Food Tech Neophobia	−0.066	0.214	0.704							
Healthiness Influence	0.217	−0.012	0.061	0.796						
Insect-Based Protein Suitability/Benefits	0.180	−0.255	−0.178	0.014	0.836					
Insect-Based Protein Willingness to Try, Buy, and Pay More	0.105	−0.284	−0.118	0.004	0.525	0.860				
Meat Nutritional Importance	−0.325	0.112	0.030	−0.189	−0.130	−0.024	0.835			
Meat Taste, Texture, Smell Importance	−0.241	−0.004	−0.004	−0.143	−0.049	0.055	0.632	0.946		
Plant-Based Protein Suitability/Benefits	0.316	−0.128	−0.098	0.174	0.201	0.047	−0.456	−0.304	0.739	
Plant-Based Protein Willingness to Try, Buy, and Pay More	0.279	−0.168	−0.120	0.205	0.184	0.181	−0.494	−0.391	0.451	0.804
**Heterotrait-Monotrait Ratio**										
Environmental Impact Influence										
Food Neophobia	0.194									
Food Tech Neophobia	0.180	0.266								
Healthiness Influence	0.317	0.171	0.124							
Insect-Based Protein Suitability/Benefits	0.241	0.296	0.219	0.078						
Insect-Based Protein Willingness to Try, Buy, and Pay More	0.154	0.334	0.145	0.064	0.586					
Meat Nutritional Importance	0.458	0.219	0.070	0.266	0.160	0.065				
Meat Taste, Texture, Smell Importance	0.313	0.180	0.048	0.180	0.060	0.079	0.729			
Plant-Based Protein Suitability/Benefits	0.434	0.217	0.166	0.217	0.240	0.083	0.559	0.328		
Plant-Based Protein Willingness to Try, Buy, and Pay More	0.398	0.281	0.180	0.288	0.239	0.280	0.644	0.457	0.563	

**Table 4:** Model Goodness of Fit (GoF) Index.

Scale	AVE	*R* ^2^	*Q^2^*(CVC)	*Q*^2^(CVR)
Insect-Based Protein Suitability/Benefits	0.699	0.042	0.532	0.027
Insect-Based Protein Willingness to Try, Buy, and Pay More	0.740	0.310	0.466	0.213
Plant-Based Protein Suitability/Benefits	0.546	0.243	0.342	0.119
Plant-Based Protein Willingness to Try, Buy, and Pay More	0.647	0.331	0.308	0.200
Average Score	0.658	0.232		4.20%
AVE × *R*^2^		0.152		
GoF = √(AVE × *R*^2^)		0.390		

**Table 5:** Direct Path Coefficients.

Hypothesized Path Relationship	Coefficient	*t*-Stat	*p*-Value
Food Neophobia → Insect-Based Protein Willingness to Try, Buy, and Pay More	−0.172	10.713	<0.001
Food Neophobia → Plant-Based Protein Willingness to Try, Buy, and Pay More	−0.089	5.195	<0.001
Food Tech Neophobia → Insect-Based Protein Willingness to Try, Buy, and Pay More	0.005	0.320	0.749
Food Tech Neophobia → Plant-Based Protein Willingness to Try, Buy, and Pay More	−0.070	4.549	<0.001
Meat Nutritional Importance → Insect-Based Protein Willingness to Try, Buy, and Pay More	0.015	0.751	0.452
Meat Nutritional Importance → Plant-Based Protein Willingness to Try, Buy, and Pay More	−0.273	12.672	<0.001
Meat Taste, Texture, Smell Importance → Insect-Based Protein Willingness to Try, Buy, and Pay More	0.067	3.272	0.001
Meat Taste, Texture, Smell Importance → Plant-Based Protein Willingness to Try, Buy, and Pay More	−0.137	6.983	<0.001
Meat Nutritional Importance → Insect-Based Protein Suitability/Benefits	−0.123	4..91	<0.001
Meat Nutritional Importance → Plant-Based Protein Suitability/Benefits	−0.379	16.505	<0.001
Meat Taste, Texture, Smell Importance → Insect-Based Protein Suitability/Benefits	0.063	2.583	0.010
Meat Taste, Texture, Smell Importance → Plant-Based Protein Suitability/Benefits	−0.007	0.309	0.757
Environmental Impact Influence → Insect-Based Protein Suitability/Benefits	0.162	7.642	<0.001
Environmental Impact Influence → Plant-Based Protein Suitability/Benefits	0.176	9.725	<0.001
Healthiness Influence → Insect-Based Protein Suitability/Benefits	−0.035	1.504	0.133
Healthiness Influence → Plant-Based Protein Suitability/Benefits	0.061	3.636	<0.001
Insect-Based Protein Suitability/Benefits → Insect-Based Protein Willingness to Try, Buy, and Pay More	0.487	38.956	<0.001
Plant-Based Protein Suitability/Benefits → Plant-Based Protein Willingness to Try, Buy, and Pay More	0.265	14.276	<0.001

**Table 6:** Plant-Based vs. Insect-Based Comparisons.

Scale Items (1 = Strongly Disagree to 5 = Strongly Agree)	Mean	*t*-Stat
19.1. Plant-based protein is healthy	4.192	35.759 *
35.1. Insect-based protein is healthy	3.432	
19.2. Plant-based protein is safe to eat	4.076	38.583 *
35.2. Insect-based protein is safe to eat	3.221	
19.3. Plant-based protein is nutritious	4.142	27.410 *
35.3. Insect-based protein is nutritious	3.555	
19.4. Plant-based protein is more sustainable	3.641	15.151 *
35.4. Insect-based protein is more sustainable	3.272	
19.5. Plant-based protein is tastier	2.645	14.236 *
35.5. Insect-based protein is tastier	2.327	
19.6. Plant-based protein is cheaper	3.253	6.795 *
35.6. Insect-based protein is cheaper	3.086	
20.1. Willing to try plant-based protein	2.633	43.130 *
36.1. Willing to try insect-based protein	1.928	
20.2. Willing to purchase plant-based protein	2.392	43.136 *
36.2. Willing to purchase insect-based protein	1.677	
20.3. Willing to pay more for plant-based protein	1.699	30.968 *
36.3. Willing to pay more for insect-based protein	1.278	

* = *p* < 0.001.
